# Improving Anxiety Related to Chronic Pain Through a Sleep Circadian Intervention Program: A Pilot Study

**DOI:** 10.3390/bs15010040

**Published:** 2025-01-03

**Authors:** Sonia López-Monzoni, Gloria Hernando Benito, Sofía Romero-Peralta, Laura Silgado-Martínez, Maria Esther Viejo-Ayuso, Leticia Álvarez-Balado, Enrique Rodríguez Matarranz, Carles Forné Izquierdo, Manuel Sánchez-de-la-Torre, Juan Fernando Masa, Ferrán Barbé, Francisco García-Río, Antonio Martínez-Nicolás, Belén García-Mediano, Esther Solano-Pérez, Olga Mediano

**Affiliations:** 1 Sleep Unit, Pneumology Department, Hospital Universitario de Guadalajara, 19002 Guadalajara, Spain; sonialopezmonzoni@gmail.com (S.L.-M.); sofiamp10@hotmail.com (S.R.-P.); unidadsuenoesther@gmail.com (M.E.V.-A.); leticia.alvarez.balado@gmail.com (L.Á.-B.); belengarmed8@gmail.com (B.G.-M.); 2 Group of Precision Medicine in Chronic Diseases, Hospital Nacional de Parapléjicos, Instituto de Investigación Sanitaria de Castilla La Mancha (IDISCAM), 45071 Toledo, Spain; 3 Medicine Department, Universidad de Alcalá, 28805 Madrid, Spain; gloria_hebe@hotmail.com; 4 Internal Medicine Department, Hospital Universitario de Guadalajara, 19002 Guadalajara, Spain; 5 Centro de Investigación Biomédica en Red de Enfermedades Respiratorias (CIBERES), 28029 Madrid, Spain; fmasa@separ.es (J.F.M.); febarbe.lleida.ics@gencat.cat (F.B.); fgr01m@gmail.com (F.G.-R.); 6 Anesthesiology Department, Hospital Universitario de Guadalajara, 19002 Guadalajara, Spain; enroma40@gmail.com; 7 Heorfy Consulting, 25007 Lleida, Spain; carles.forne@heorfy.com; 8 Department of Basic Medical Sciences, Universitat de Lleida, 25198 Lleida, Spain; 9 Department of Nursing, Physiotherapy and Occupational Therapy, Faculty of Physiotherapy and Nursing, Universidad de Castilla la Mancha, 45071 Toledo, Spain; 10 Respiratory Department, Hospital Universitario San Pedro Alcántara, 10003 Cáceres, Spain; 11 Translational Research in Respiratory Medicine, Hospital Universitari Arnau de Vilanova-Santa Maria, Biomedical Research Institute of Lleida (IRBLleida), 25198 Lleida, Spain; 12 Medicine Department, Universidad Autónoma de Madrid, 28049 Madrid, Spain; 13 Respiratory Department, Hospital Universitario La Paz, Instituto de Investigación Hospital Universitario La Paz (IdiPaz), 28046 Madrid, Spain; 14 Chronobiology Lab, Department of Physiology, College of Biology, Universidad de Murcia, Mare Nostrum Campus, IUIE, IMIB-Arrixaca, 30100 Murcia, Spain; antilas@um.es; 15 Human Physiology Area, Faculty of Sport Sciences, Universidad de Murcia, Santiago de La Ribera-San Javier, 30720 Murcia, Spain; 16 Centro de Investigación Biomédica en Red de Fragilidad y Envejecimiento Saludable (CIBERFES), 28029 Madrid, Spain

**Keywords:** chronic pain, sleep, sleep disorders, circadian rhythm, anxiety, depression, opioids, drugs consumption

## Abstract

The limitations of pharmacological treatments for chronic pain have become increasingly evident: dependency, side effects, resistance, and diminishing efficacy. The urgent need for innovative solutions has become a compelling focus for improving patient outcomes. Innovative non-pharmacological approaches, such as sleep management, as a strategy to reduce opioid consumption and pain control are needed. The aim was to evaluate the impact of a Sleep and Circadian Intervention Program (SCIP) in the control of chronic musculoskeletal pain (CMP). This was a randomized clinical trial (NCT03646084), in which 49 CMP patients were assigned to SCIP (*n* = 15, mean age 51 years and 40% women) or non-SCIP groups (*n* = 26, 53 years and 61.5% women). Outcomes were evaluated after 6 months through self-reported questionnaires (pain intensity, physical function, depression/anxiety, and quality of life (QoL)). The SCIP group was assessed by polysomnography and specific questionnaires and was treated for diagnosed sleep disorders according to clinical guidelines. This population showed a moderate pain intensity at baseline, important deterioration of QoL and pathological anxiety/fear related to pain. Fifty percent of them presented restless leg syndrome, 60% moderate/severe insomnia, and 62.5% sleep apnea. During the follow-up, the SCIP group presented a greater reduction in the abnormal risk group for anxiety (from 73.3% to 46.7%) and depression (from 53.3% to 33.3%) on the Hospital Anxiety and Depression Scale compared to the non-SCIP group (from 40% to 29.2% and 33.3% to 29.2%, respectively). Also, a positive significant effect on anxiety/fear related to pain was found in the Pain Anxiety Symptoms Scale multivariable model, with an important improvement in symptoms. The application of SCIP in CMP patients improved anxiety and controlled associated sleep disorders, highlighting the impact on insomnia. Larger studies are needed for better understanding of the sleep intervention in CMP control.

## 1. Introduction

### 1.1. Chronic Pain: Definition, Consequences, and Management

Chronic musculoskeletal pain (CMP) is a frequent condition defined as uncontrolled pain for at least three months (Perrot et al., 2019). It is composed of a complex interaction among psychological, physiological, circadian, and social factors with considerable quality of life (QoL) impairment (Perrot et al., 2019). Consequently, it has resulted in a major healthcare problem related to treatment cost, absenteeism, disability, and drug consumption (Dueñas et al., 2016; Schultz & Licciardone, 2022) and has become a first order social and economic problem around the world (Lentz et al., 2020; Mills et al., 2019).

CMP control includes drug prescriptions that can end in excessive opioids consumption, which is an increasing health problem according to the Substance Abuse and Mental Health Services Administration (SAMHSA) reports since 2014 (https://www.samhsa.gov/data/nsduh/national-releases, accessed on 29 December 2024 (NSDUH National Releases, 2022)). In addition, drug abuse has had an impact on the increasing number of deaths over the years (Curtin et al., 2023). For these reasons, actions to reduce and control the excessive and questionable use of opioids are needed. Strategies to achieve these objectives have been initiated, some of them successfully (Dart et al., 2015; Neuman et al., 2019), but these are still not enough.

### 1.2. Chronic Pain and Sleep Relationship

Because of the persistence of pain over a long period of time, the sleep quality of patients may be variable and disturbed. Sleep disturbances can be present, comprising insomnia, drug-related sleep disorders, sleep-disordered breathing (SDB), or restless legs syndrome (RLS), among others (Ferini-Strambi, 2017; Luo et al., 2022; Ma et al., 2014; Tang et al., 2007). It has been observed that CMP patients have a decrease in overall sleep time, with 42% of patients sleeping less than six hours, which predisposes patients to have a decrease in the algesia threshold (Artner et al., 2012).

Also, a strong association was found between the presence of sleep circadian disorders (SCDs) and the elevated risk of suffering from CMP. In fact, prevalence of SCDs ranges from 50% to 80% in patients with CMP, and SCD severity is related to pain intensity (Cheatle et al., 2016). Therefore, these SCDs, which are usually undiagnosed and untreated, contribute to reinforcing the negative impact of CMP (Cheatle et al., 2016). A study pointed to a 74.4% prevalence of SDB in patients with a specific subtype of CMP, chronic low back pain (CLBP) (Steinmetz et al., 2023). Also, obstructive sleep apnea (OSA) was related to different types of CMP, such as pain related to psoriasis, chronic craniofacial pain, chronic widespread pain, and fibromyalgia (Li et al., 2022). Insomnia and its symptoms were associated with the development of pain over a 12-month period (Roth, 2007; Canivet et al., 2008); and narcolepsy, which seemed to have a higher frequency of CMP in comparison to control subjects (Cremaschi et al., 2019). Finally, it has been shown that sleep deprivation produces hyperalgesic changes (Koffel et al., 2016).

Mechanisms that link SCDs and CMP control have been proposed (Li et al., 2022). Different neurotransmitters and neuroendocrine substances have been implicated in the mediation of CMP and SCDs, suggesting complex pathways governing both entities. As an example, sleep deprivation can interfere with analgesic treatments involving opioidergic and serotoninergic mechanisms of action (Frohnhofen, 2018; Haack et al., 2020; Lautenbacher et al., 2006).

Therefore, the presence of CMP can be the cause of SCDs and, on the other hand, can be the result of them making it difficult to control. A bidirectional relationship has been established between sleep quality and pain intensity. Specifically, the quality of sleep was observed to diminish after days of intense pain, while an increase in pain intensity was noted on the day following a lack of sleep (Alsaadi et al., 2011). This suggests that ensuring good sleep quality is essential for pain control and medication reduction (Bascour-Sandoval et al., 2021; Koffel et al., 2016).

However, CMP is a complex condition with multiple aspects implicated on its intensity. Depression and anxiety, potentially modifiable pain-related disorders, are the most frequent psychiatric illnesses in CMP patients with a prevalence of 55% and 49%, respectively (Sagheer et al., 2013). Whether these symptoms are due to SCD or CMP or to comorbid depression/anxiety is difficult to discern (Sun et al., 2021). CMP places patients at greater risk of experiencing associated symptoms such as an inability to concentrate, a pessimistic mood, fatigue, and a loss of motivation, which may give rise to adverse influences on a patient’s treatment and recovery process (Hong et al., 2014).

Another important aspect is the impaired QoL (Bentsen et al., 2008; Semeru & Halim, 2019). Patients with CLBP have an elevated functional disability assessed through the 36-item Short Form Health Survey (SF-36) (Hong et al., 2014). Some determinants such as the psychological/physical state of the patient and social factors could be transcendental in the QoL of these patients. Their identification allows us to apply strategies for stress reduction, CMP management and QoL improvement (Agnus Tom et al., 2022). Considering not only the physical but also the psychological and emotional implications of CMP, some studies have shown that pain acceptance strategies could have a positive impact on the QoL of these patients (Poppe et al., 2011; Semeru & Halim, 2019).

This evidence, therefore, highlights the need for comprehensive management strategies (Cheatle & Webster, 2015; Tong et al., 2024). It has been shown that non-pharmacological sleep interventions may represent a fruitful avenue for optimizing treatment outcomes (Tang et al., 2015). However, there is a lack of evidence on the impact of sleep management as a strategy to reduce drug consumption and pain control (Craige et al., 2023; Murawski et al., 2018). Innovative non-pharmacological approaches to this end are needed (Aronowitz et al., 2021; Kundakci et al., 2022) and a better understanding of modifiable disorders such as SCDs will help in directing more effective treatments.

We conducted a randomized clinical trial (RCT) to evaluate the effectiveness of managing SCDs in people with CMP. We hypothesize that a sleep circadian intervention program (SCIP) could be effective in improving pain control outcomes and drug use in patients suffering from CMP. For a complete understanding about the impact of the intervention, the improvement of the following related issues was evaluated: (1) pain perception and pain intensity control; (2) QoL; (3) mood and anxiety; (4) physical functioning; (5) pharmacological consumption.

## 2. Materials and Methods

### 2.1. Study Design

This was a pilot study, multicenter randomized clinical trial (RCT) (National Clinical Trial 03646084) to evaluate the impact of a SCIP on opioid consumption and pain control outcomes. The study was developed in the sleep units of the Hospital Universitario de Guadalajara (Guadalajara), Hospital Universitario La Paz (Madrid) and Hospital San Pedro de Alcántara (Cáceres), Spain. Participants were randomized (1:1) to either the intervention (SCIP) or non-SCIP group, with stratification based on exposure to opioid treatment, conducted in blocks of twelve participants at each site.

Subjects had to be older than 18 years old. Patients with severe psychopathological comorbidity, malignant/terminal diseases, shift-workers, or pregnant women were excluded. All patients provided written consent prior to the inclusion in the study. The study was approved by the Ethics and Clinical Trials Committee (2018.11.PR).

### 2.2. Procedures

**Figure 1:**
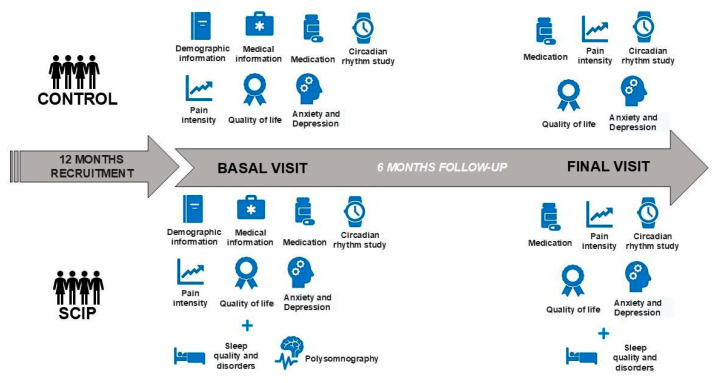
Methodological diagram of tests conducted in control and Sleep Circadian Intervention Program (SCIP) groups at baseline and 6-month follow-up visits.

The study had a total duration of 18 months (12 months of recruitment and 6 months of follow-up) and all interventions included in the study methodology were carried out on the participants (Figure 1).

#### 2.2.1. Basal Visit

For both SCIP and non-SCIP groups, various clinical, pain and demographic information was collected. The following variables were recorded: age, sex (demographic characteristics), comorbidities (clinical information), weight, height, body mass index (BMI), waist and hip circumference, blood pressure, heart rate, oxygen saturation (anthropometric data), time and cause of pain (pain characteristics).

Pharmacologic sleep- and pain-related agents and dosing were carefully recorded, including active ingredient, route of administration, dose, schedule, start and end date. In the case of opioids, the equivalent dose of opioids was used, calculated with the tables of opioid equivalents, based on the idea that doses of different opioids produce a similar analgesic effect (equianalgesic doses) (Dowell et al., 2022; Nielsen et al., 2016).

For a comprehensive effect of the overall the complexity of CMP and the benefit of the SCIP, diverse functional aspects were evaluated as follows:

(1) Pain intensity evaluation and control was evaluated by the Numerical Rating Scale (NRS), where patients rate their pain on a scale from 0 (“no pain”) to 10 (“worst pain”). A more detailed description of the questionnaires used and its interpretation and the variables of the study are described in Appendix A.1 and Appendix A.2.
(2) General QoL was measured through the 36-item Short Form Health Survey (SF-36) and the EQ-5D five-level version (EQ-5D-5L), and for the SCIP, the Functional Outcomes of Sleep Questionnaire (FOSQ) was administered.

SF-36 evaluates physical functioning, role limitations (physical and emotional), pain, general health, vitality (energy/fatigue), social functioning, and mental health. Higher scores indicate better health or functioning on a scale from 0 to 100.

EQ-5D-5L measures mobility, self-care, usual activities, pain/discomfort, and anxiety/depression. Each dimension is rated on a 5-level scale, ranging from “no problems” to “unable/extreme problems”.

The FOSQ evaluates activity level, vigilance, intimacy and sexual relationships, general productivity, and social outcomes. Higher scores indicate better functional outcomes.

(3) Mood and anxiety were evaluated through the Hospital Anxiety and Depression Scale (HADS). It consists of 14 items, divided equally between two subscales: anxiety and depression. Each item is scored on a 4-point scale, with subscale scores ranging from 0 to 21. Higher scores indicate greater symptom severity.

The specific impact of pain on anxiety was assessed through the Pain Anxiety Symptoms Scale (PASS-20), assessing cognitive anxiety, escape/avoidance, fear, and physiological anxiety. Each item is rated on a 6-point scale, with higher scores indicating greater pain-related anxiety.

The non-SCIP group received pain management according to current clinical practice. The SCIP group underwent sleep questionnaires and polysomnography (PSG) for sleep disorders diagnosis. When a sleep disorder was identified, specific sleep interventions were implemented following clinical guidelines. Also, pain management was applied as in the non-SCIP group. This intervention in the SCIP group could be summarized as follows:

(1) Educational and circadian rhythm intervention: general sleep hygiene recommendations and physical activity promotion based on the circadian rhythm study results.
(2) Assessment of sleep quality and pharmacological and non-pharmacological treatment of sleep disorders detected, through:
  (2.1) Full PSG, which was performed and scored according to the American Academy of Sleep Medicine (AASM) (Kapur et al., 2017). OSA was considered when the apnea–hypopnea index (AHI) was above 15/h, moderate between 15 and 30/h, and severe with AHI above 30/h.
  (2.2) Specific questionnaires for sleep disorders, including: subjective quality of sleep: Pittsburgh Sleep Quality Index (PSQI) (global score ranging from 0 to 21; higher scores indicate poorer sleep quality). Daytime hypersomnolence: Epworth Sleepiness Scale (ESS) (total score ranges from 0 to 24, with higher scores indicating greater levels of daytime sleepiness). Insomnia: Insomnia Severity Index (ISI): total score ranges from 0 to 28. Scores are categorized as follows: absence of insomnia (0–7), subclinical insomnia (8–14), moderate clinical insomnia (15–21), and severe clinical insomnia (22–28). The ISI is widely used for both clinical evaluation and monitoring treatment outcomes. Diagnosis was made on the basis of the presence of all of five clinical symptoms following the Restless legs syndrome (RLS) criteria. Diagnosis was stabilized following the standard criteria established by the International Restless Legs Syndrome Study Group.

Therapeutic intervention according to current guidelines for the management of sleep disorders and considering the following recommendations: (1) Control of SDB including OSA (including continuous positive airway pressure, adaptive servo-ventilation and bi-level positive airway pressure ventilation for central sleep apnea if needed). (2) Attempt to improve anxiety, depression, and insomnia with pharmacological/non-pharmacological action taken by the sleep specialist criteria trying to avoid the use of benzodiazepines, sedatives, and hypnotics. (3) Treatment of RLS, if needed, following the treatment guidelines. (4) Pain control: opioid dose reduction and trying non-opioid therapies (e.g., non-steroidal anti-inflammatory drugs [NSAIDs], antiepileptic drugs, physical therapy, antidepressants) instead of opioids. (5) Caution against alcohol use.

#### 2.2.2. Follow-Up

After 6 months, the measurements were repeated in both groups and the functional improvement was assessed. The non-SCIP group was followed by their specialist doctor in chronic pain, who indicated the necessary treatments to control CMP, in accordance with current clinical guidelines. In the intervention group, the SCIP protocol was also applied by the research team.

The impact of the SCIP was evaluated based on the improvement of the following aspects: (1) pain perception and pain intensity control; (2) QoL; (3) mood and anxiety; (4) physical function; and (5) changes in pharmacological consumption (equivalent dose of opioids).

### 2.3. Statistical Analysis

Data description was performed using frequency and percentage for categorical variables and mean and standard deviation (SD) for continuous variables. No imputation method was used to supply missing data. The Pearson χ2 test and the t test were used for unadjusted comparisons between the study arms.

To assess differences in the evolution of outcomes between the intervention and non-SCIP groups, multivariable mixed-effects regression models were fitted. Gamma distribution was assumed for opioids equivalent dose, while Gaussian distribution was assumed for Health Utility Index, and beta distribution was assumed for bounded outcomes, dividing by their ranges to obtain values between 0 and 1, i.e., NRS, SF-36 total score, FOSQ total score, HADS anxiety and depression, PASS-20 total score. Independent variables included baseline outcome value and the interaction between months of follow-up and the study arm. The model for the opioid equivalent dose also included baseline non-opioid and adjuvant analgesic treatments. The statistically significant effect of the interaction term was assessed by comparing the fitted model with the model without interaction by means of the likelihood ratio test (significant results means differential evolution between study arms).

The pre-post comparison of the specific questionnaires administered to the SCIP group was carried out using the McNemar test for dichotomous variables, the McNemar–Bowker test for categorical variables of more than two categories, and the paired t test for continuous variables.

All statistical tests were two-sided, and the significance level (α) was set at 0.05. Analysis was performed with the R statistical package version 4.3.1 (R Foundation for Statistical Computing).

## 3. Results

### 3.1. Population Characteristics

**Figure 2:**
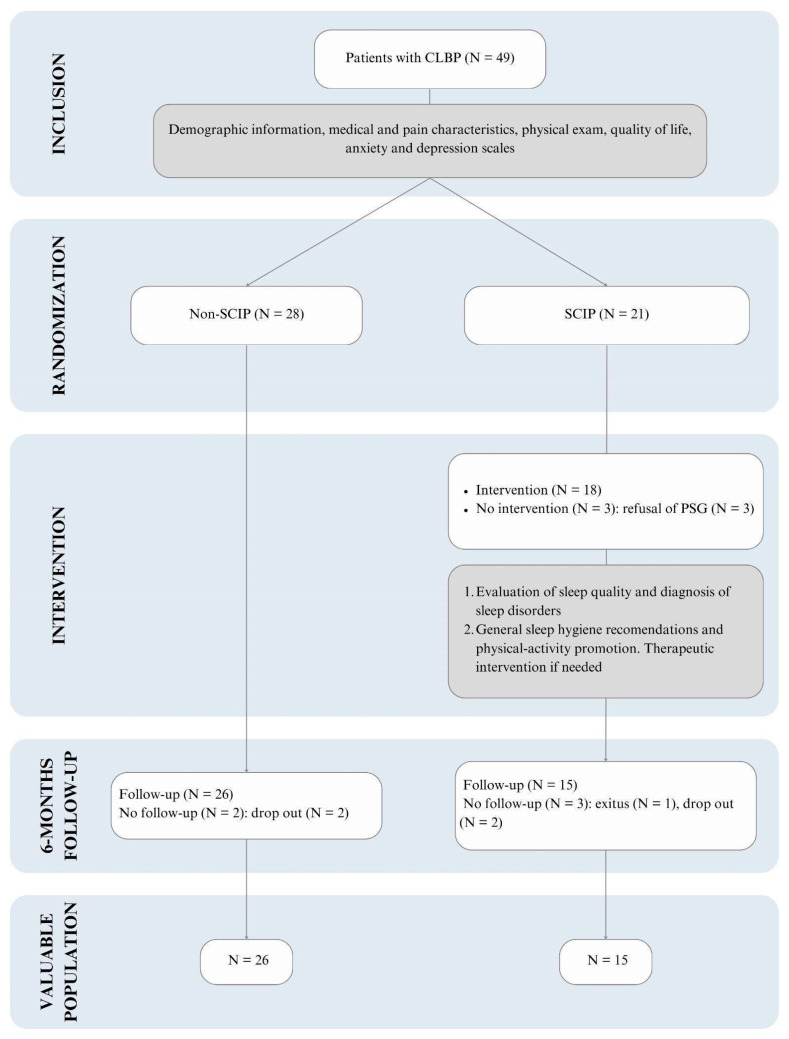
Flow chart of inclusion and exclusion for patients. CLBP: chronic low back pain. SCIP: Sleep and Circadian Intervention Program. PSG: polysomnography.

Forty-nine patients, referred to treatment for CLBP lasting more than three months, were recruited and randomized in the three participant sites, resulting in 21 in the SCIP group and 28 in the non-SCIP group. In the SCIP group, three patients did not undergo the intervention due to their refusal to perform the sleep study. Of those who received the intervention, three were lost before the follow-up (one due to death and two dropped out from the study). In the non-SCIP group, two were lost before the follow-up. Consequently, 15 SCIP and 26 controls were analyzed (Figure 2).

Demographic and pain characteristics of the valuable population (*n* = 41) are summarized in Table 1. General characteristics, underlying medical pathologies or the usual medication for pain control were reasonably well-balanced between the study groups. The mean (SD) age was 51 (7) years in the SCIP group and 53 (11) in the non-SCIP group, with 40% and 61.5% of the total being women, respectively. The mean duration of CMP was over a year for most of the patients in the SCIP and non-SCIP groups (100% and 92.0%, respectively), and the most frequent medication for pain relief was conventional analgesics (93.3% and 95.7%, respectively), followed by opioids (100% and 87.0%, respectively), and antidepressants (73.3% and 47.8%, respectively).

### 3.2. Opioids Consumption

At baseline, opioid consumption was 75.1 (73.3) mg/day in the SCIP group and 37.5 (40.0) mg/day in the non-SCIP. At the follow-up visit, the SCIP group reported an opioid consumption of 70.1 (63.3) mg/day vs. 34.4 (41.1) mg/day in the non-SCIP group (Table 2).

### 3.3. Pain Related Outcomes Measured by Questionnaires Results

Measured by the NRS, patients presented a moderate pain intensity at baseline with a mean (SD) score of 6.64 (2.02) in the SCIP group vs. 6.50 (1.86) in the non-SCIP. In the follow-up visit, NRS score was 5.79 (2.01) and 5.80 (2.16), respectively.

When the impact of QoL on global evaluation was measured by the SF-36, the perception of the state of health was negatively affected in both groups, at baseline. The evolution of the QoL after the 6-month follow-up was not significantly different between the SCIP and non-SCIP groups. The results of the EQ-5D-5L showed an impact on mobility, self-care, usual activities, pain/discomfort, and anxiety and depression in both groups, with a health utility index of 0.47 (0.26) vs. 0.52 (0.21) corresponding with an important deterioration of the self-perceived QoL. The evolution of this index over time was 0.59 (0.18) vs. 0.62 (0.23). In the FOSQ, it was observed that sleepiness affected activities of daily living in both groups, with a subtle improvement at the end of the study.

In the SCIP group, 73.3% of the patients showed abnormal anxiety values and 53.3% abnormal depression values vs. 40.0% and 33.3% in the non-SCIP group. There was a reduction in the total number of patients in the abnormal risk group in anxiety (SCIP (46.7%) and non-SCIP (29.2%)) and depression (33.3 vs. 29.2%) after the follow-up.

The PASS-20 scale showed that patients often experienced high levels of anxiety and fear related to pain intensity in both the SCIP (57.7 (19.3)) and non-SCIP (57.5 (23.8)) groups, with a reduction at the end of the study visit: 47.4 (19.7) vs. 55.7 (20.9).

There were no differences of clinical significance in the intensity of pain, QoL, and anxiety or depression characteristics at baseline between the groups (Table 3).

### 3.4. Polysomnographic Characteristics in the Intervention Group

Sleep architecture was analyzed in the SCIP group (Table 4), showing a sleep efficiency of 79.5 (19.0%), with an increased sleep latency (67 (107) min), and a greatly increased REM (rapid eye movement) latency (172 (103) min). Related to sleep architecture, there was a predominance of light sleep (N1 + N2: 68.9%) with 17.3% (18.0) of sleep time in deep sleep and decreased REM sleep (11.8 (8.40) %). An increased sleep fragmentation was shown when analyzed with a wakefulness after sleep onset (WASO) of 39.3 min and an arousal index of 25.9 (15.2) was recorded.

The mean (SD) global apnea–hypopnea index (AHI) was 26.6 (23.9) events per hour, being 62.5% of the participants diagnosed with OSA (37.5% severe OSA). Related to the events distribution, a predominance of obstructive events and events in supine position was shown, expending a mean of 64.8% of the time in this position.

### 3.5. Sleep Questionnaires in the Intervention Group

The results of the sleep questionnaires evaluated in the SCIP group are presented in Table 5. Related to the RLS, 50% of patients presented a positive result at baseline. An insomnia diagnosis was established using the well-validated ISI questionnaire with a mean value of 16.3 (5.1), indicating the presence of clinical insomnia and showing 60% of patients in the moderate/severe group. In the PSQI, the mean (SD) score was 14.90 (3.66), which indicated clinical sleep impairment and worse sleep quality, with “sleep difficulties” results in almost all the items. Daytime sleepiness was measured by the ESS, showing a mean value of 10 (5), indicating mild excessive daytime sleepiness.

In the analysis of the evolution of the diagnosed sleep pathologies (Table 5), patients identified as positive by the RLS questionnaire decreased to 8.3% after the intervention, although the difference was not statistically significant. An improvement was observed in the ISI questionnaire, which significantly changed from moderate clinical insomnia to below those pathological levels. Furthermore, the percentage of patients belonging to the moderate–severe insomnia group decreased to 46.6% after the intervention. No clinically relevant differences were found in either PSQI or ESS results.

### 3.6. Effectiveness of the Intervention

The effectiveness of the intervention was assessed using multivariable mixed-effects regression models between the intervention and non-SCIP groups, for opioid equivalent doses and questionnaire scores.

The multivariable model of opioid dose consumption between the study groups showed no differences in evolution (*p* value for interaction = 0.825) (Table A1).

**Figure 3:**
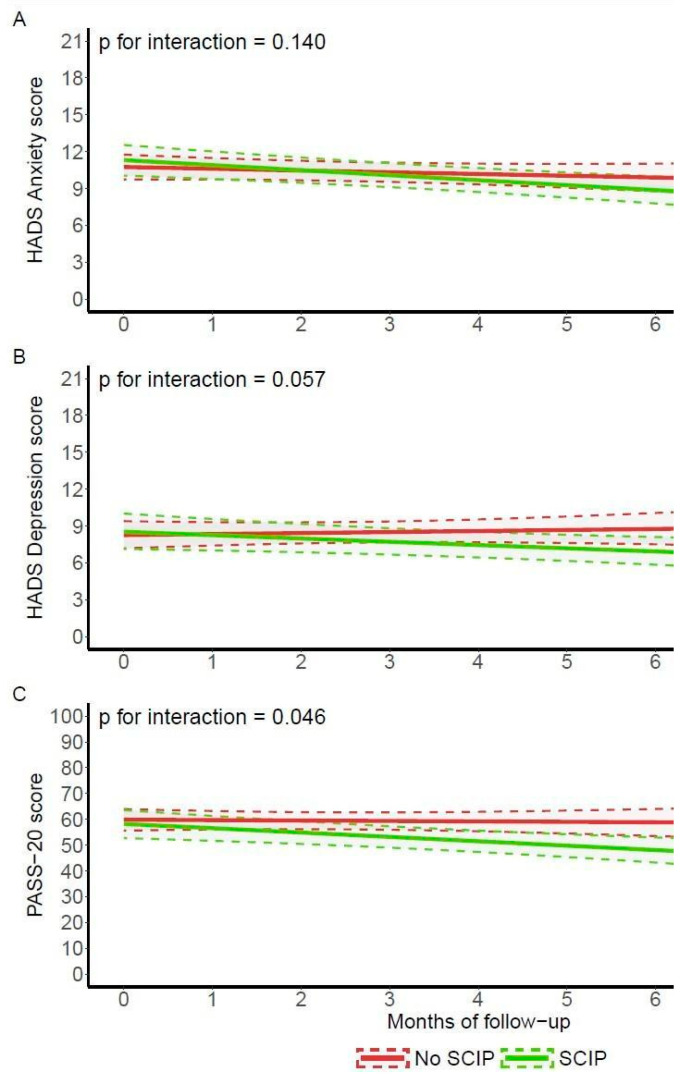
Graphical representation of longitudinal model fit for HADS anxiety and depression, and PASS-20 score trajectory. (**A**) Results of the multivariable model of the evolution of the HADS anxiety score between the study groups. The evolution of the HADS score was not statistically different between groups for anxiety (*p*-value for the interaction = 0.140). (**B**) Results of the multivariable model of the evolution of the HADS depression score between the study groups. The evolution was not statistically significant (*p*-value for the interaction = 0.057). (**C**) Evolution of the PASS-20 total score between study groups. PASS-20 score was statistically different between groups (*p* value for interaction = 0.046). Abbreviations: HADS: hospital anxiety and depression scale. SCIP: sleep circadian intervention program. PASS-20: pain anxiety symptoms scale.

In relation to the assessment of pain intensity using the NRS, no statistically significant differences were found between groups (Table A2). No statistically significant differences were found in QoL related to the intervention either in the EQ-5D-5L (Table A3), SF36 (Table A4) or the FOSQ (Table A5) questionnaires. The evolution of the HADS anxiety score was not statistically different between groups (Figure 3A and Table A6), and neither was the depression score (Figure 3B and Table A7).

A positive, statistically significant effect of SCIP on anxiety or fear of patients in relation to pain was found in the multivariable model of the evolution of the PASS-20 score (parameter (95% CI) −0.0613 (−0.128; 0.00514)) (*p* value for interaction = 0.046) (Figure 3C and Table A8).

## 4. Discussion

This pilot study analyzed the feasibility of a sleep management protocol in the improvement of patients with CLBP, as a non-pharmacological measure, and its impact on the main spectrums that comprise it. In terms of feasibility, the protocol has demonstrated a satisfactory level of adaptation, albeit with a non-negligible rate of attrition, slightly higher than desirable, particularly within the SCIP group. This may likely be attributed to the complexity of the protocol, suggesting a consideration for its simplification to facilitate replication in a larger population.

No differences were found in the evolution of drug consumption, including opioids. We did not expect to find an impact at this level, as it was a pilot study. However, it does seem feasible that this can be maintained as the main objective for the design of the definitive study.

Related to the global impact of moderate intensity CLBP, lasting more than a year in most cases, it has been shown how this population frequently presents a great deterioration in relevant spheres, such as QoL, high levels of anxiety, and depression and functional impairment. These results highlight that the management of CLBP does not have a unidirectional meaning, but rather requires the comprehensive management of the patient with relevant interactions between the different aspects that comprise it.

On the other hand, it has been important to confirm the association of CLBP with short sleep duration and poor sleep quality, with consistent data previously reported (Murase et al., 2015). The use of PSG confirmed a slight decrease in sleep efficiency, prolonged latencies, a relevant sleep fragmentation and WASO accompanied by a predominance of superficial sleep. In addition, the frequent presence of various sleep disorders such as insomnia, OSA, or RLS was confirmed, indicating the importance of performing a sleep evaluation in the CLBP population. These findings are consistent with data from the literature and confirm an increased risk for sleep disturbances after experiencing CLBP (Axén, 2016). Previous results, such as those shown by the National Health and Nutrition Examination Survey (NHANES) (Tong et al., 2024), also found a notable prevalence of sleep disorders in patients with CLBP. Given the relationship between sleep quality and pain, the need for sleep management as part of a global pain management strategy was suggested by Alsadi et al. (Alsaadi et al., 2011). For instance, the application of the SCIP allowed the diagnosis of important unnoticed SCDs.

It is interesting to highlight some relevant issues about the presence of respiratory events, with more than half of the patients presenting an AHI higher than 15/h. It would be expected that the predominance of these respiratory events was of central origin and with signs of hypoventilation, caused by central nervous system depressant substance intake (Walker et al., 2007). However, it is surprising that most of the respiratory events were from an obstructive origin with oxygen and transcutaneous capnography in normal values, showing a positive diagnosis of OSA in these patients, being moderate-to-severe in most cases. In this sense, this could be conditioned, in part, by the position adopted by CLBP patients. In this study, it has been confirmed that most of these patients spent most of their time in a supine position, which clearly favors this type of event.

The frequent occurrence of RLS (Baykal Şahin et al., 2023) conditioned by pain itself was previously reported, as well as the higher prevalence of insomnia (Asih et al., 2014), induced both by local pathology at the lower back level and by the associated medication (antidepressants). Recent studies have found a prevalence of RLS of 33.5% in patients with CLBP (Baykal Şahin et al., 2023; Luo et al., 2022), in line with our findings. It may have important relevance not only in the knowledge of common pathophysiological conditions but also in the holistic approach to both problems due to the negative impact on QoL. In addition, our study population showed a frequent presence of moderate/severe symptoms. The global ISI improved significantly after the sleep program, changing from values compatible with clinical insomnia (>15) to being below that threshold. In addition, a reduction in the percentage of patients belonging to the moderate–severe insomnia severity group was seen, with an increase in the patients with absence of insomnia symptoms. This improvement could have a relevant clinical impact, as previous reports showed a potential bidirectional causal association between genetically predicted insomnia and CMP (Luo et al., 2022). The subjective perception of sleep quality through the PSQI was also impaired in these patients with CLBP, in a similar way to that previously shown by Steinmetz A et al. (Steinmetz et al., 2023).

Regarding the impact of the intervention, it is worth highlighting the effectiveness in the management of most cases of OSA, RLS, and insomnia identified, but none for either PSQI or daytime hypersomnolence measured by the ESS. Therefore, it has addressed pathologies with a great impact on both QoL and pain control that would otherwise have remained unidentified. Accordingly, Pakpour et al., in an observational cohort study of 761 patients with CLBP, showed that sleep disturbance was a pain intensity risk factor that decreased after the sleep disorders were resolved (Pakpour et al., 2018).

The most important finding of our study is the improvement in anxiety levels of patients in the SCIP group. When anxiety levels were measured using the HADS, greater improvements were found in the SCIP group versus non-SCIP. These improvements could have clinical relevance, since the change in the overall score in the SCIP group went from a high risk of anxiety to categories of possible anxiety. In addition, a decrease in the number of patients located in the significant anxiety group was found, with an increase in those that did not present an anxiety risk. This improvement is more evident in the evolution of the PASS-20 questionnaire, reflecting less anxiety due to the fear triggered by the presence of pain. This positive evolution throughout the follow-up showed a significant improvement in all the analyzed areas (cognitive anxiety responses, escape and avoidance, fearful thinking, and physiological anxiety responses) in the SCIP group. It would be of interest to analyze these results in a greater population to confirm these findings. While no statistically significant differences were observed in HADS anxiety scores in our study, the PASS-20 did show significant improvements, suggesting that the SCIP effects are more pronounced in areas closely linked to pain-related anxiety. PASS-20 specifically evaluates anxiety related to the experience of pain, whereas the HADS anxiety score evaluates general anxiety, which may not have been as directly affected by the intervention.

The observed improvements in anxiety and depression in the SCIP group could be attributed to the management of sleep disorders. Poor sleep quality is well-documented to exacerbate both anxiety and depression, as it may disrupt emotion regulation and stress (O’Leary et al., 2017). By improving sleep quality, SCIP may reduce the underlying contributors to anxiety and depression. Additionally, the SCIP may reduce the burden of CMP. Improved sleep often enhances pain perception and reduces the bidirectional relationship of pain-induced anxiety and sleep disruption (Andersen et al., 2018). Consequently, this intervention program could create a synergistic effect, promoting both psychological and physical relief. Nevertheless, the improvements observed in the non-SCIP group might reflect a natural progression of the condition, or the influence of other factors such as social support, routine care, or the Hawthorne effect, defined as decreased symptom expression due to being observed (Berthelot et al., 2019). However, the changes observed in the SCIP compared to non-SCIP groups suggest that the sleep interventions may amplify these effects.

Some study limitations need to be commented on. First, although it was a 1:1 randomized study, groups were slightly imbalanced, which was because the block size was too large. An alternative could be to define smaller blocks, since the balance between the groups would be better controlled. In addition, there were high losses before the follow-up visit of the intervention group, which could be related to the study design. Although it is a well-designed study that demonstrated a satisfactory adaptation, its complexity could condition the follow-up of the participants. A suggestion for a larger-scale study could be to consider performing a home sleep apnea test (HSAT) instead of a PSG, as the latter was the main limitation of the intervention group. This could also avoid the one-night effect associated with PSG and, considering that information from a circadian rhythm study is available, simplified tests could be an adequate alternative. The heterogeneity of the groups in this study represents an important factor to consider when interpreting the results. Variability in baseline characteristics such as pain severity, sleep quality, and psychological status could have influenced the observed outcomes. However, the heterogeneity observed in the groups is justified by the inherently diverse nature of patients with CMP in clinical practice. These patients often present with varying degrees of pain severity, sleep disturbances or emotional comorbidities, reflecting the complex nature of chronic pain. This variability mirrors real-world clinical settings, making the findings more applicable to everyday clinical scenarios. Future research could focus on stratifying patients who may benefit most from interventions such as SCIP, optimizing personalized treatment strategies.

The strengths of the study comprise its multicenter design and the complete diagnosis of sleep disorders performed by sleep-medicine experts. Secondly, pain management was addressed from a comprehensive point of view, with systematic collection through validated questionnaires of all the areas that comprise it. In addition, this study identified potential issues related to the study design. It aimed to develop an innovative and profitable model to achieve a comprehensive, accurate, and personalized management of chronic pain care. In addition, in a larger cohort study, it could contribute to the resolution of problems in the field of Public Health and in clinical practice, and it could include the study of the circadian rhythm disorders in a specific population.

## 5. Conclusions

The SCIP may have positive effects in patients with CMP, reducing anxiety and improving the control of sleep disorders, especially the impact on insomnia. More clinical trials are needed to assess the feasibility of the implementation in clinical practice of these insights of the management of CMP. A better understanding of modifiable disturbances such as sleep disorders will help in directing a more effective pain treatment and rehabilitation.

## Figures and Tables

**Table 1:** Demographic, clinical and pain characteristics of the valuable population.

	SCIP (*n* = 15)	Non-SCIP (*n* = 26)	*p* Value
Demographic characteristics			
Age [years]	51 (7)	53 (11)	0.496
Women	6 (40%)	16 (61.5%)	0.424
Anthropometric data			
Body mass index [kg/m^2^]	28 (4)	27 (3)	0.274
Mean systolic BP [mmHg]	120 (15)	123 (15)	0.534
Mean diastolic BP [mmHg]	80 (10)	80 (9)	0.889
Pain and sleep medication			
Any medication	15 (100%)	23 (92.0%)	0.515
Opioids	15 (100%)	20 (87.0%)	0.277
Benzodiazepines	7 (46.7%)	6 (26.1%)	0.338
Melatonin	0 (0%)	1 (4.35%)	>0.999
Antidepressant	11 (73.3%)	11 (47.8%)	0.222
Anticonvulsant	0 (0%)	1 (4.35%)	>0.999
Antihistamine	0 (0%)	2 (8.7%)	0.503
Comorbidities			
Hypertension	5 (33.3%)	4 (15.4%)	0.245
Diabetes mellitus	1 (6.67%)	2 (7.69%)	>0.999
Anxiety-depressive syndrome	6 (40.0%)	6 (23.1%)	0.296
Kidney failure	0 (0%)	1 (3.85%)	>0.999
Vascular disease	3 (21.4%)	3 (11.5%)	0.653
Other relevant background	13 (86.7%)	23 (88.5%)	>0.999
Cause of pain			0.656
Discopathy	13 (86.7%)	19 (82.6%)	
Arthrosis	0 (0%)	2 (8.70%)	
Other causes	2 (13.3%)	2 (8.70%)	
Time with pain			
More than 3 months	0 (0%)	0 (0%)	
More than 6 months	0 (0%)	2 (8.00%)	
More than 1 year	15 (100%)	23 (92.0%)	

Frequencies and percentages for categorical variables. Mean (SD) for continuous variables. SCIP: Sleep and Circadian Intervention Program.

**Table 2:** Concomitant medication at baseline and follow-up in the valuable population.

	Basal Visit			Follow-Up Visit		
	SCIP (*n* = 15)	Non-SCIP (*n* = 26)	*p* Value	SCIP (*n* = 15)	Non-SCIP (*n* = 26)	*p* Value
Pain and sleep medication						
Equivalent dose of opioids [mg/day]	75.1 (73.3)	37.5 (40.0)	0.082	70.1 (63.3)	34.4 (41.4)	0.065
Non opioids—analgesic	13 (86.7%)	21 (84.0%)	>0.999	14 (93.3%)	20 (80.0%)	0.383
Adjuvants	14 (93.3%)	19 (73.0%)	0.220	14 (93.3%)	19 (73.0%)	0.220

Frequencies and percentages for categorical variables. Mean (SD) for continuous variables. SCIP: Sleep and Circadian Intervention Program.

**Table 3:** Summary of the questionnaire scores and comparability between the groups in the basal and end of study visits.

Questionnaire Scores	Basal Visit			Follow-Up Visit		
	SCIP (*n* = 15)	Non-SCIP (*n* = 26)	*p* Value	SCIP (*n* = 15)	Non-SCIP (*n* = 26)	*p* Value
Pain intensity						
NRS	6.64 (2.02)	6.50 (1.86)	0.83	5.79 (2.01)	5.80 (2.16)	0.98
Quality of life						
SF-36						
Physical function	48.0 (21.7)	42.9 (23.2)	0.48	48.0 (16.5)	51.5 (20.8)	0.57
Physical role function	20.0 (38.0)	16.3 (30.0)	0.75	16.7 (29.4)	42.0 (41.9)	0.03 *
Emotional role function	51.1 (46.9)	46.2 (43.3)	0.74	53.3 (46.8)	66.7 (43.0)	0.38
Energy/fatigue	27.3 (20.7)	38.8 (17.3)	0.08	32.7 (16.0)	39.2 (13.6)	0.20
Emotional well-being	54.4 (18.4)	60.0 (21.4)	0.38	55.7 (21.2)	58.9 (19.5)	0.64
Social function	47.5 (25.1)	56.2 (30.1)	0.33	45.8 (24.4)	62.0 (24.1)	0.05
Pain	24.7 (16.1)	30.7 (17.3)	0.27	28.7 (19.2)	39.7 (21.9)	0.11
General health	38.7 (19.3)	39.8 (15.3)	0.83	43.7 (17.7)	38.6 (10.8)	0.33
Health change	38.3 (32.6)	36.5 (25.7)	0.86	46.7 (28.1)	40.0 (23.9)	0.45
SF-36 total score	40.9 (19.6)	41.6 (18.2)	0.91	42.5 (16.6)	49.9 (15.3)	0.18
EQ-5D-5L						
Mobility						
No limitation	4 (26.7%)	2 (7.69%)		3 (20.0%)	7 (28.0%)	
Mild limitation	3 (20.0%)	10 (38.5%)		3 (20.0%)	7 (28.0%)	
Moderate limitation	6 (40.0%)	9 (34.6%)		9 (60.0%)	8 (32.0%)	
Severe limitation	2 (13.3%)	5 (19.2%)		0 (0%)	3 (12.0%)	
Cannot walk	0 (0%)	0 (0%)		0 (0%)	0 (0%)	
Self-care						
No limitation	5 (33.3%)	10 (38.5%)		5 (33.3%)	12 (48.0%)	
Mild limitation	6 (40.0%)	7 (26.9%)		7 (46.7%)	8 (32.0%)	
Moderate limitation	4 (26.7%)	9 (34.6%)		3 (20.0%)	4 (16.0%)	
Severe limitation	0 (0%)	0 (0%)		0 (0%)	1 (4.0%)	
Cannot wash or dress themselves	0 (0%)	0 (0%)		0 (0%)	0 (0%)	
Daily life activities			0.03 *			0.66
No limitation	1 (6.7%)	0 (0%)		1 (6.67%)	3 (12.0%)	
Mild limitation	1 (6.7%)	11 (42.3%)		3 (20.0%)	9 (36.0%)	
Moderate limitation	8 (53.3%)	12 (46.2%)		9 (60.0%)	10 (40.0%)	
Severe limitation	3 (20.0%)	3 (11.5%)		2 (13.3%)	2 (8.0%)	
Cannot do usual activities	2 (13.3%)	0 (0%)		0 (0%)	1 (4.0%)	
Pain						
No limitation	1 (6.7%)	0 (0%)		0 (0%)	0 (0%)	
Mild limitation	0 (0%)	0 (0%)		1 (6.7%)	4 (16.0%)	
Moderate limitation	4 (26.7%)	12 (46.2%)		7 (46.7%)	12 (48.0%)	
Severe limitation	6 (40.0%)	13 (50.0%)		7 (46.7%)	8 (32.0%)	
Extreme pain or discomfort	4 (26.7%)	1 (3.9%)		0 (0%)	1 (4.0%)	
Anxiety and depression			0.24			
No limitation	3 (20.0%)	8 (30.8%)		6 (40.0%)	11 (44.0%)	
Mild limitation	3 (20.0%)	9 (34.6%)		5 (33.3%)	7 (28.0%)	
Moderate limitation	8 (53.3%)	5 (19.2%)		2 (13.3%)	7 (28.0%)	
Severe limitation	1 (6.7%)	3 (11.5%)		2 (13.3%)	0 (0%)	
Extremely anxious or depressed	0 (0%)	1 (3.9%)		0 (0%)	0 (0%)	
Health utility index	0.47 (0.26)	0.52 (0.21)	0.57	0.59 (0.18)	0.62 (0.23)	0.63
FOSQ						
Total score	13.60 (3.59)	14.70 (3.46)	0.39	14.80 (3.50)	15.60 (2.35)	0.44
Anxiety and depression						
HADS						
Anxiety	11.5 (4.4)	8.6 (4.6)	0.06	9.2 (3.9)	8.2 (3.6)	0.43
Depression	9.9 (4.8)	7.5 (4.9)	0.14	8.7 (5.2)	7.5 (4.2)	0.46
Anxiety, risk groups			0.11			0.31
-Normal [0–7]	3 (20.0%)	12 (48.0%)		7 (46.7%)	11 (45.8%)	
-Borderline abnormal [8–10]	1 (6.7%)	3 (12.0%)		1 (6.7%)	6 (25.0%)	
-Abnormal [11–21]	11 (73.3%)	10 (40.0%)		7 (46.7%)	7 (29.2%)	
Depression, risk groups			0.40			>0.99
-Normal [0–7]	4 (26.7%)	12 (50.0%)		6 (40.0%)	11 (45.8%)	
-Borderline abnormal [8–10]	3 (20.0%)	4 (16.7%)		4 (26.7%)	6 (25.0%)	
-Abnormal [11–21]	8 (53.3%)	8 (33.3%)		5 (33.3%)	7 (29.2%)	
PASS-20						
Cognitive	19.4 (5.1)	17.0 (5.7)	0.17	16.2 (5.4)	16.0 (5.4)	0.91
Escape/avoid	15.6 (6.2)	16.7 (5.9)	0.57	14.4 (5.2)	16.2 (5.2)	0.31
Fear	12.2 (6.5)	12.5 (7.6)	0.89	8.6 (6.3)	12.2 (7.0)	0.10
Physiological anxiety	10.6 (6.5)	11.2 (7.7)	0.78	8.2 (6.8)	11.3 (6.0)	0.16
PASS-20 total score	57.8 (19.3)	57.5 (23.8)	0.96	47.4 (19.7)	55.7 (20.9)	0.22

Categorical variables are presented as n (%) and continuous variables are represented as mean (SD). SCIP: sleep circadian intervention program; NRS: Numerical Rating Scale; SF-36: 36-Item Short Form Survey; EQ-5D-5L: EQ-5D five-level version. FOSQ: Functional Outcomes of Sleep Questionnaire. HADS: Hospital Anxiety and Depression Scale. PASS-20: Pain Anxiety Symptoms Scale. * Statistically significance.

**Table 4:** Polysomnographic characteristics in the intervention group.

Sleep architecture	
TST [min]	376 (58)
Sleep efficiency [% of TST]	79.5 (19.0)
Sleep latency [min]	67 (107)
REM latency [min]	172 (103)
WASO [min]	39.3 (24.4)
N1 sleep time [% of TST]	16.8 (14.4)
N2 sleep time [% of TST]	52.1 (16.7)
N3 sleep time [% of TST]	17.3 (18.0)
Total REM sleep [% of TST]	11.8 (8.4)
Arousal index [/h]	
Total	25.9 (15.2)
Respiratory	17.2 (15.3)
Spontaneous	7.8 (5.5)
Legs	0 (0)
Other	0.9 (0.7)
Apneas—hypopneas index [events/h] and saturation parameters	
Mixed	7.8 (17.1)
Central	1.7 (4.5)
Obstructive	1.8 (3.2)
Hypopneas	16.8 (15.0)
Supine AHI	36.2 (28.3)
Supine time [%]	64.8 (31.7)
Non supine AHI	36.2 (28.3)
Global AHI	26.6 (23.9)
Desaturation index	11.1 (15.0)
T90 [%]	9.0 (14.2)
OSA diagnosis (AHI ≥ 15/h)	10 (62.5%)
Moderate OSA (15/h ≤ AHI)	4 (25.0%)
Severe OSA (AHI ≥ 30/h)	6 (37.5%)

Categorical variables are presented as n (%) and continuous variables are represented as mean (SD). TST: total sleep time; REM: rapid eye movement; WASO: wakefulness after sleep onset; AHI: apnea-hypopnea index; T90: percentage of time with an oxygen saturation under 90%. OSA: obstructive sleep apnea.

**Table 5:** Sleep questionnaires result in the SCIP group at baseline and follow-up visit.

Sleep Questionnaires Result in SCIP	N	Basal Visit	Follow-Up Visit	*p*-Value
Restless Legs Syndrome	12			
Positive result		6 (50.0%)	1 (8.3%)	0.074
ISI	15			
Insomnia severity index		16.3 (5.1)	13.5 (6.2)	0.033 *
Severity groups				
Absence of clinical insomnia [0–7]		0 (0%)	4 (26.7%)	
Subclinical insomnia [8–14]		6 (40.0%)	4 (26.7%)	
Clinical insomnia (moderate) [15–21]		7 (46.7%)	6 (40.0%)	
Clinical insomnia (severe) [22–28]		2 (13.3%)	1 (6.67%)	
PSQI	14			
Subjective sleep quality		2.14 (0.53)	1.71 (0.91)	0.105
Sleep latency		1.79 (1.19)	1.79 (1.19)	>0.999
Sleep duration		2.50 (0.94)	2.21 (0.89)	0.265
Usual sleep efficiency		1.86 (1.29)	2.14 (0.95)	0.430
Sleep disturbances		2.14 (0.53)	2.00 (0.55)	0.484
Use of hypnotic medication		2.21 (1.31)	1.93 (1.38)	0.423
Daytime dysfunction		2.21 (0.89)	1.71 (0.91)	0.098
Total score		14.90 (3.66)	13.50 (4.33)	0.191
Epworth	15			
Total score		10 (5)	8 (4)	0.220

Categorical variables are presented as n (%) and continuous variables are represented as mean (SD). SCIP: sleep circadian intervention program; ISI: Insomnia Severity index; PSQI: Pittsburgh Sleep Quality Index. * Statistically significance.

## Data Availability

The original contributions presented in this study are included in the article. Further inquiries can be directed to the corresponding authors.

## Appendix A. Detailed Description of Questionnaires Used and Its Application

### Appendix A.1. Pain Perception and Pain Intensity Control

- Numerical Rating Scale (NRS) is a commonly used tool for evaluating intensity, with reference to pain experience over the past week in a 10 cm horizontal scale. A higher score indicates a greater pain intensity categorized as: none (0), mild (1–3), moderate (4–6), and severe (7–10).

### Appendix A.2. Quality of Life

- 36-Item Short Form Survey (SF-36) is composed of 36 items that assess health states and cover the following scales: physical function, physical role, body pain, general health, vitality, social function, emotional role and mental health. Each scale is directly transformed into a 0–100 scale on the assumption that each question carries equal weight. The lower the score, the more disability.
- EQ-5D five-level version (EQ-5D-5L): the patient themselves assesses their state of health in five levels of severity (from 1: no problems to 5: severe problems and extreme problems) by five dimensions (descriptive system): mobility, self-care, usual activities, pain/discomfort and anxiety/depression (Stolk et al., 2019). For the analysis, the health utility index was used, which indicates the patient’s self-perceived quality of life (the index ranges between a value of 1: best health and 0: death). A utility value was assigned to each unique combination of responses on the questionnaire. The utility values of each dimension were then added to obtain the total health utility index for an individual. References for the Spanish population have been used to establish the categories when performing the calculation (Ramos-Goñi et al., 2018).
- Functional Outcomes of Sleep Questionnaire (FOSQ) (Weaver et al., 1997) was designed to assess the impact of sleep on various aspects of the patient’s life and its functional impact. It is a 30-item questionnaire with five categories: activity level, vigilance, intimacy and sexual relationships, general productivity, social outcome, and difficulty in performing a given activity. Each item has four possible answers: “no difficulty”, “little difficulty”, “moderate difficulty”, and “a lot of difficulty”. The scale of items is four to six points for each item. The total score equals the mean scores for each subscale multiplied by the number of subscales answered, being the total score between 5 and 20, with higher scores indicating better functional status (Ferrer et al., 1999).

### Appendix A.3. Mood and Anxiety

- Hospital Anxiety and Depression Scale (HADS) (Herrero et al., 2003; Turk et al., 2015) is a self-report questionnaire that measures the overall severity of anxiety and depression. It is composed of a 14-item questionnaire, 7 for anxiety and 7 for depression, each rated on a four-point scale (0–3) and a total score per subscale that ranges from 0 to 21. A total score of 0–7 is considered normal, 8–10 is suggestive of possible anxiety/depression, and >10 is indicative of mood disorder or disease.
- Pain Anxiety Symptoms Scale (PASS20) (López-Martínez et al., 2021) is a short self-report questionnaire, a version of the Pain Anxiety Symptoms Scale (PASS) (McCracken & Dhingra, 2002), that measures fear and anxiety responses to specific experiences of chronic or recurrent pain. It is composed of 20 items, each rated on a five-point scale (from 0: never to 5: always). It consists of four subscales measuring cognitive anxiety responses, escape and avoidance, fearful thinking, and physiological anxiety responses. It ranges from a score of 0 to 100, where 0 corresponds to the absence of symptoms and scores above 80 correspond to the presence of almost constant symptoms.

### Appendix A.4. Other Sleep Disorders

- Restless legs syndrome (RLS) (Walters et al., 1995): diagnosis was made on the basis of the presence of all of five clinical symptoms following the RLS diagnostic criteria. Diagnosis was stabilized following the standard criteria established by the International Restless Legs Syndrome Study Group (Allen et al., 2014).
- Insomnia Severity Index (ISI) (Bastien, 2001): insomnia was defined as the inability to acquire adequate sleep related to difficulties initiating and/or maintaining sleep and the feeling of absence of rest during the following day. To determine the presence of clinical insomnia, patients completed the well-validated ISI questionnaire, which includes seven items (rated on a scale from 0 to 4) for assessing perceived insomnia severity. Total score ranges from 0 to 28 (with higher scores indicative of more severe insomnia) and suggesting clinical insomnia if ISI ≥ 15.
- Pittsburgh Sleep Quality Index (PSQI) (Buysse et al., 1989): sleep measurement about multiple sleep domains evaluating sleep impairment and sleep quality. It consists of 19 questions (scores ranging from 0 to 21) about sleep habits and measures sleep quality, sleep latency, sleep duration, habitual efficiency, disturbances, use of sleep medications, and daytime dysfunction. A score > 5 indicates clinical sleep impairment and higher scores reveal worse sleep quality.
- Epworth Sleepiness Score (ESS) (Johns, 1991): used to assess daytime sleepiness. It helps to evaluate how likely an individual is to doze off or fall asleep in various situations during the day. The ESS consists of eight scenarios, and for each one, the person rates their likelihood of falling asleep on a scale from 0 to 3. The total score ranges from 0 to 24 with the following interpretation: 1 to 6 points: normal sleepiness, 7 to 8 points: moderate sleepiness, 9 to 24 points: abnormal sleepiness (possibly pathological).

## Appendix B. Study Variables and Data Collection

Data collection was carried out in a database created for this purpose, where the variables included in the study were registered and stored.

Basic demographic information, medical characteristics (age [year], sex [woman/man], job, medication) and pain characteristics (pain cause, time, location, and intensity) were collected.

In the physical examination, the following data were recorded: weight (kilograms), height (centimeters), body mass index (BMI) (kilograms per square meter), neck, hip, waist circumference (centimeters), and blood pressure (millimeters of mercury).

Pharmacologic sleep and pain agents and dosing were carefully recorded, including: opioid analgesics, benzodiazepine receptor agonists, non-benzodiazepine receptor agonists, melatonin receptor agonists, antidepressants, antipsychotics, anticonvulsants, general analgesics, and antihistamines.

Polysomnographic variables: total sleep time (minutes), sleep efficiency (percentage of total sleep time over total recording time), no REM latency (minutes), REM latency (minutes), WASO (percentage of total sleep time), N1 sleep time (percentage of total sleep time), N2 sleep time (percentage of total sleep time), N3 sleep time (percentage of total sleep time), total REM sleep (percentage of total sleep time), arousal (total index, respiratory index, spontaneous index, legs index), apneas—hypopneas (total index, mixed index, central index, obstructive index, hypopneas index, supine and non-supine index) and saturation parameters (desaturation index, CT90: time desaturation below 90%).

**Table A1:** Marginal effects on the longitudinal evolution of opioid doses.

Factor	Parameter (95% CI)	Standard Error	*p* Value
Intercept	2.85 (1.71; 3.99)	0.582	<0.001
SCIP (vs. non-SCIP)	0.045 (−1.23; 1.32)	0.652	0.945
Months of follow-up	−0.00501 (−0.17; 0.16)	0.084	0.953
Basal Opioid Dose	0.0145 (0.00522; 0.0238)	0.005	0.002
Basal non-opioid analgesics (vs. No)	−0.202 (−1.49; 1.09)	0.660	0.760
Basal adjuvant treatment (vs. No)	−0.549 (−1.81; 0.715)	0.645	0.395
Months: group interaction (SCIP vs. non-SCIP)	0.0297 (−0.259; 0.318)	0.147	0.840

**Table A2:** Marginal effects on the longitudinal evolution of the NRS pain score.

Factor	Parameter (95% CI)	Standard Error	*p* Value
Intercept	−2.02 (−2.67; −1.37)	0.333	<0.001
SCIP (vs. non-SCIP)	0.194 (−0.254; 0.641)	0.228	0.397
Months of follow-up	−0.0362 (−0.0884; 0.0161)	0.027	0.175
Basal NRS score	0.406 (0.31; 0.502)	0.049	<0.001
Months: group interaction (SCIP vs. non-SCIP)	0.0256 (−0.0684; 0.12)	0.048	0.593

**Table A3:** Marginal effects on the longitudinal evolution of the EQ-5D-5L health utility.

Factor	Parameter (95% CI)	Standard Error	*p* Value
Intercept	0.0709 (0.00955; 0.132)	0.031	0.029
SCIP (vs. non-SCIP)	−0.00819 (−0.07; 0.0536)	0.032	0.796
Months of follow-up	0.0156 (0.00674; 0.0245)	0.004	0.001
EQ-5D-5L baseline utility	0.866 (0.771; 0.96)	0.048	<0.001
Months: group interaction (SCIP vs. non-SCIP)	0.00159 (−0.0119; 0.0151)	0.007	0.818

**Table A4:** Marginal effects on the longitudinal evolution of the SF-36 total score.

Factor	Parameter (95% CI)	Standard Error	*p* Value
Intercept	−1.83 (−2.08; −1.58)	0.128	<0.001
SCIP (vs. non-SCIP)	−0.0223 (−0.268; 0.224)	0.125	0.859
Months of follow-up	0.0483 (0.0124; 0.0841)	0.018	0.008
SF-36 basal score	0.036 (0.0313; 0.0406)	0.002	<0.001
Months: group interaction (SCIP vs. non-SCIP)	−0.0384 (−0.0919; 0.0151)	0.027	0.160

**Table A5:** Marginal effects on the longitudinal evolution of the FOSQ total score.

Factor	Parameter (95% CI)	Standard Error	*p* Value
Intercept	−3.3 (−4; −2.59)	0.360	<0.001
SCIP (vs. Non-SCIP)	0.0197 (−0.376; 0.416)	0.202	0.922
Months of follow-up	0.0708 (0.00786; 0.134)	0.032	0.027
FOSQ basal score	0.268 (0.22; 0.316)	0.024	<0.001
Months: group interaction (SCIP vs. non-SCIP)	−0.00446 (−0.0937; 0.0847)	0.046	0.922

**Table A6:** Marginal effects on the longitudinal evolution of the HADS anxiety score.

Factor	Parameter (95% CI)	Standard Error	*p* Value
Intercept	−2.08 (−2.4; −1.77)	0.160	<0.001
SCIP (vs. Non-SCIP)	0.106 (−0.195; 0.408)	0.154	0.489
Months of follow-up	−0.0273 (−0.074; 0.0194)	0.024	0.252
HADS basal score for anxiety	0.194 (0.167; 0.221)	0.014	<0.001
Months: group interaction (SCIP vs. non-SCIP)	−0.0506 (−0.115; 0.0143)	0.033	0.127

**Table A7:** Marginal effects on the longitudinal evolution of the HADS depression score.

Factor	Parameter (95% CI)	Standard Error	*p* Value
Intercept	−2.49 (−2.84; −2.15)	0.176	<0.001
SCIP (vs. Non-SCIP)	0.0541 (−0.298; 0.407)	0.180	0.763
Months of follow-up	0.0164 (−0.0393; 0.0721)	0.028	0.564
HADS basal score for depression	0.229 (0.197; 0.26)	0.016	<0.001
Months: group interaction (SCIP vs. non-SCIP)	−0.0713 (−0.146; 0.00371)	0.038	0.062

**Table A8:** Marginal effects on the longitudinal evolution of the PASS-20 score.

Factor	Parameter (95% CI)	Standard Error	*p* Value
Intercept	−1.94 (−2.3; −1.58)	0.184	<0.001
SCIP (vs. Non-SCIP)	−0.0674 (−0.365; 0.23)	0.152	0.657
Months of follow-up	−0.00685 (−0.053; 0.0392)	0.024	0.771
PASS-20 basal score	0.0403 (0.0343; 0.0462)	0.003	<0.001
Months: group interaction (SCIP vs. non-SCIP)	−0.0613 (−0.128; 0.00514)	0.034	0.071

## References

[B1-behavsci-15-00040] Agnus Tom A., Rajkumar E., John R., Joshua George A. (2022). Determinants of quality of life in individuals with chronic low back pain: A systematic review. Health Psychology and Behavioral Medicine.

[B2-behavsci-15-00040] Allen R. P., Picchietti D. L., Garcia-Borreguero D., Ondo W. G., Walters A. S., Winkelman J. W., Zucconi M., Ferri R., Trenkwalder C., Lee H. B. (2014). Restless legs syndrome/Willis–Ekbom disease diagnostic criteria: Updated International Restless Legs Syndrome Study Group (IRLSSG) consensus criteria—History, rationale, description, and significance. Sleep Medicine.

[B3-behavsci-15-00040] Alsaadi S. M., McAuley J. H., Hush J. M., Maher C. G. (2011). Prevalence of sleep disturbance in patients with low back pain. European Spine Journal.

[B4-behavsci-15-00040] Andersen M. L., Araujo P., Frange C., Tufik S. (2018). Sleep disturbance and pain: A tale of two common problems. Chest.

[B5-behavsci-15-00040] Aronowitz S. V., Compton P., Schmidt H. D. (2021). Innovative approaches to educating future clinicians about opioids, pain, addiction and health policy. Pain Management Nursing: Official Journal of the American Society of Pain Management Nurses.

[B6-behavsci-15-00040] Artner J., Cakir B., Spiekermann J. A., Kurz S., Leucht F., Reichel H., Lattig F. (2012). Prevalence of sleep deprivation in patients with chronic neck and back pain: A retrospective evaluation of 1016 patients. Journal of Pain Research.

[B7-behavsci-15-00040] Asih S., Neblett R., Mayer T. G., Brede E., Gatchel R. J. (2014). Insomnia in a chronic musculoskeletal pain with disability population is independent of pain and depression. The Spine Journal.

[B8-behavsci-15-00040] Axén I. (2016). Pain-related sleep disturbance: A prospective study with repeated measures. The Clinical Journal of Pain.

[B9-behavsci-15-00040] Bascour-Sandoval C., Belmar-Arriagada H., Albayay J., Lacoste-Abarzua C., Bielefeldt-Astudillo D., Gajardo-Burgos R., Vidal-Torres M., Gálvez-García G. (2021). The effect of sleep quality on pain in chilean individuals with musculoskeletal disorders. International Journal of Environmental Research and Public Health.

[B10-behavsci-15-00040] Bastien C. (2001). Validation of the insomnia severity index as an outcome measure for insomnia research. Sleep Medicine.

[B11-behavsci-15-00040] Baykal Şahin H., Karacaoğlu S., Çapkın E., Kara F. (2023). Restless legs syndrome in patients with chronic low back pain. British Journal of Pain.

[B12-behavsci-15-00040] Bentsen S. B., Hanestad B. R., Rustøen T., Wahl A. K. (2008). Quality of life in chronic low back pain patients treated with instrumented fusion. Journal of Clinical Nursing.

[B13-behavsci-15-00040] Berthelot J. M., Nizard J., Maugars Y. (2019). The negative Hawthorne effect: Explaining pain overexpression. Joint Bone Spine.

[B14-behavsci-15-00040] Buysse D. J., Reynolds C. F., Monk T. H., Berman S. R., Kupfer D. J. (1989). The Pittsburgh sleep quality index: A new instrument for psychiatric practice and research. Psychiatry Research.

[B15-behavsci-15-00040] Canivet C., Ostergren P.-O., Choi B., Nilsson P., Sillén U. A., Moghadassi M., Karasek R., Isacsson S.-O. (2008). Sleeping problems as a risk factor for subsequent musculoskeletal pain and the role of job strain: Results from a one-year follow-up of the malmö shoulder neck study cohort. International Journal of Behavioral Medicine.

[B16-behavsci-15-00040] Cheatle M. D., Webster L. R. (2015). Opioid therapy and sleep disorders: Risks and mitigation strategies. Pain Medicine.

[B17-behavsci-15-00040] Cheatle M. D., Foster S., Pinkett A., Lesneski M., Qu D., Dhingra L. (2016). Assessing and managing sleep disturbance in patients with chronic pain. Anesthesiology Clinics.

[B18-behavsci-15-00040] Craige E. A., Memon A. R., Belavy D. L., Vincent G. E., Owen P. J. (2023). Effects of non-pharmacological interventions on sleep in chronic low back pain: A systematic review and meta-analysis of randomised controlled trials. Sleep Medicine Reviews.

[B19-behavsci-15-00040] Cremaschi R. C., Hirotsu C., Tufik S., Coelho F. M. (2019). Chronic pain in narcolepsy type 1 and type 2—An underestimated reality. Journal of Sleep Research.

[B20-behavsci-15-00040] Curtin S. C., Tejada-Vera B., Bastian B. A. (2023). Deaths: Leading Causes for 2020. National Vital Statistics Reports.

[B21-behavsci-15-00040] Dart R. C., Severtson S. G., Green J. L. (2015). Abuse-deterrent formulations of prescription opioids. JAMA Psychiatry.

[B22-behavsci-15-00040] Dowell D., Ragan K. R., Jones C. M., Baldwin G. T., Chou R. (2022). CDC clinical practice guideline for prescribing opioids for pain—United States, 2022. MMWR Recommendations and Reports.

[B23-behavsci-15-00040] Dueñas M., Ojeda B., Salazar A., Mico J. A., Failde I. (2016). A review of chronic pain impact on patients, their social environment and the health care system. Journal of Pain Research.

[B24-behavsci-15-00040] Ferini-Strambi L. (2017). Neuropathic pain and sleep: A review. Pain and Therapy.

[B25-behavsci-15-00040] Ferrer M., Vilagut G., Monasterio C., Montserrat J. M., Mayos M., Alonso J. (1999). Measurement of the perceived impact of sleep problems: The Spanish version of the functional outcomes sleep questionnaire and the Epworth sleepiness scale. Medicina Clinica.

[B26-behavsci-15-00040] Frohnhofen H. (2018). Pain and sleep. Zeitschrift Für Gerontologie Und Geriatrie.

[B27-behavsci-15-00040] Haack M., Simpson N., Sethna N., Kaur S., Mullington J. (2020). Sleep deficiency and chronic pain: Potential underlying mechanisms and clinical implications. Neuropsychopharmacology.

[B28-behavsci-15-00040] Herrero M. J., Blanch J., Peri J. M., De Pablo J., Pintor L., Bulbena A. (2003). A validation study of the hospital anxiety and depression scale (HADS) in a Spanish population. General Hospital Psychiatry.

[B29-behavsci-15-00040] Hong J. H., Kim H. D., Shin H. H., Huh B. (2014). Assessment of depression, anxiety, sleep disturbance, and quality of life in patients with chronic low back pain in Korea. Korean Journal of Anesthesiology.

[B30-behavsci-15-00040] Johns M. W. (1991). A new method for measuring daytime sleepiness: The epworth sleepiness scale. Sleep.

[B31-behavsci-15-00040] Kapur V. K., Auckley D. H., Chowdhuri S., Kuhlmann D. C., Mehra R., Ramar K., Harrod C. G. (2017). Clinical practice guideline for diagnostic testing for adult obstructive sleep apnea: An American academy of sleep medicine clinical practice guideline. Journal of Clinical Sleep Medicine.

[B32-behavsci-15-00040] Koffel E., Kroenke K., Bair M. J., Leverty D., Polusny M. A., Krebs E. E. (2016). The bidirectional relationship between sleep complaints and pain: Analysis of data from a randomized trial. Health Psychology.

[B33-behavsci-15-00040] Kundakci B., Kaur J., Goh S. L., Hall M., Doherty M., Zhang W., Abhishek A. (2022). Efficacy of nonpharmacological interventions for individual features of fibromyalgia: A systematic review and meta-analysis of randomized controlled trials. Pain.

[B34-behavsci-15-00040] Lautenbacher S., Kundermann B., Krieg J.-C. (2006). Sleep deprivation and pain perception. Sleep Medicine Reviews.

[B35-behavsci-15-00040] Lentz T. A., Rhon D. I., George S. Z. (2020). Predicting opioid use, increased health care utilization and high costs for musculoskeletal pain: What factors mediate pain intensity and disability?. The Journal of Pain.

[B36-behavsci-15-00040] Li M. T., Robinson C. L., Ruan Q. Z., Surapaneni S., Southerland W. (2022). The influence of sleep disturbance on chronic pain. Current Pain and Headache Reports.

[B37-behavsci-15-00040] Luo G., Yao Y., Tao J., Wang T., Yan M. (2022). Causal association of sleep disturbances and low back pain: A bidirectional two-sample mendelian randomization study. Frontiers in Neuroscience.

[B38-behavsci-15-00040] López-Martínez A. E., Esteve R., Ruiz-Párraga G. T., Rueda-Serrano C., Serrano-Ibáñez E. R., Ramírez-Maestre C. (2021). Psychometric properties of the Spanish version of the Pain Anxiety Symptoms Scale-20 (PASS-20-SV). Psicothema.

[B39-behavsci-15-00040] Ma C.-L., Chang W.-P., Lin C.-C. (2014). Rest/activity rhythm is related to the coexistence of pain and sleep disturbance among advanced cancer patients with pain. Supportive Care in Cancer.

[B40-behavsci-15-00040] McCracken L. M., Dhingra L. (2002). A short version of the Pain Anxiety Symptoms Scale (PASS-20): Preliminary development and validity. Pain Research and Management.

[B41-behavsci-15-00040] Mills S. E. E., Nicolson K. P., Smith B. H. (2019). Chronic pain: A review of its epidemiology and associated factors in population-based studies. BJA: British Journal of Anaesthesia.

[B42-behavsci-15-00040] Murase K., Tabara Y., Ito H., Kobayashi M., Takahashi Y., Setoh K., Kawaguchi T., Muro S., Kadotani H., Kosugi S., Sekine A., Yamada R., Nakayama T., Mishima M., Matsuda S., Matsuda F., Chin K. (2015). Knee pain and low back pain additively disturb sleep in the general population: A cross-sectional analysis of the nagahama study. PLoS ONE.

[B43-behavsci-15-00040] Murawski B., Wade L., Plotnikoff R. C., Lubans D. R., Duncan M. J. (2018). A systematic review and meta-analysis of cognitive and behavioral interventions to improve sleep health in adults without sleep disorders. Sleep Medicine Reviews.

[B44-behavsci-15-00040] Neuman M. D., Bateman B. T., Wunsch H. (2019). Inappropriate opioid prescription after surgery. Lancet.

[B45-behavsci-15-00040] Nielsen S., Degenhardt L., Hoban B., Gisev N. (2016). A synthesis of oral morphine equivalents (OME) for opioid utilisation studies. Pharmacoepidemiology and Drug Safety.

[B46-behavsci-15-00040] NSDUH National Releases (2022). Substance abuse and mental health services administration.

[B47-behavsci-15-00040] O’Leary K., Bylsma L. M., Rottenberg J. (2017). Why might poor sleep quality lead to depression? A role for emotion regulation. Cognition & Emotion.

[B48-behavsci-15-00040] Pakpour A. H., Yaghoubidoust M., Campbell P. (2018). Persistent and developing sleep problems: A prospective cohort study on the relationship to poor outcome in patients attending a pain clinic with chronic low back pain. Pain Practice.

[B49-behavsci-15-00040] Perrot S., Cohen M., Barke A., Korwisi B., Rief W., Treede R. D., IASP Taskforce for the Classification of Chronic Pain (2019). The IASP classification of chronic pain for ICD-11: Chronic secondary musculoskeletal pain. Pain.

[B50-behavsci-15-00040] Poppe C., Crombez G., Devulder J., Hanoulle I., Vogelaers D., Petrovic M. (2011). Personality traits in chronic pain patients are associated with low acceptance and catastrophizing about pain. Acta Clinica Belgica.

[B51-behavsci-15-00040] Ramos-Goñi J. M., Craig B. M., Oppe M., Ramallo-Fariña Y., Pinto-Prades J. L., Luo N., Rivero-Arias O. (2018). Handling data quality issues to estimate the Spanish EQ-5D-5L value set using a hybrid interval regression approach. Value in Health.

[B52-behavsci-15-00040] Roth T. (2007). Insomnia: Definition, prevalence, etiology, and consequences. Journal of Clinical Sleep Medicine.

[B53-behavsci-15-00040] Sagheer M. A., Khan M. F., Sharif S. (2013). Association between chronic low back pain, anxiety and depression in patients at a tertiary care centre. JPMA The Journal of the Pakistan Medical Association.

[B54-behavsci-15-00040] Schultz M. J., Licciardone J. C. (2022). The effect of long-term opioid use on back-specific disability and health-related quality of life in patients with chronic low back pain. Journal of Osteopathic Medicine.

[B55-behavsci-15-00040] Semeru G. M., Halim M. S. (2019). Acceptance versus catastrophizing in predicting quality of life in patients with chronic low back pain. The Korean Journal of Pain.

[B56-behavsci-15-00040] Steinmetz A., Hacke F., Delank K.-S. (2023). Pressure pain thresholds and central sensitization in relation to psychosocial predictors of chronicity in low back pain. Diagnostics.

[B57-behavsci-15-00040] Stolk E., Ludwig K., Rand K., Van Hout B., Ramos-Goñi J. M. (2019). Overview, update, and lessons learned from the international EQ-5D-5L valuation work: Version 2 of the EQ-5D-5L valuation protocol. Value in Health.

[B58-behavsci-15-00040] Sun Y., Laksono I., Selvanathan J., Saripella A., Nagappa M., Pham C., Englesakis M., Peng P., Morin C. M., Chung F. (2021). Prevalence of sleep disturbances in patients with chronic non-cancer pain: A systematic review and meta-analysis. Sleep Medicine Reviews.

[B59-behavsci-15-00040] Tang N. K. Y., Lereya S. T., Boulton H., Miller M. A., Wolke D., Cappuccio F. P. (2015). Nonpharmacological treatments of insomnia for long-term painful conditions: A systematic review and meta-analysis of patient-reported outcomes in randomized controlled trials. Sleep.

[B60-behavsci-15-00040] Tang N. K. Y., Wright K. J., Salkovskis P. M. (2007). Prevalence and correlates of clinical insomnia co-occurring with chronic back pain. Journal of Sleep Research.

[B61-behavsci-15-00040] Tong Y., Zhang X.-Q., Zhou H.-Y. (2024). Chronic low back pain and sleep disturbance in adults in the US: The NHANES 2009–2010 Study. Pain Physician.

[B62-behavsci-15-00040] Turk D. C., Dworkin R. H., Trudeau J. J., Benson C., Biondi D. M., Katz N. P., Kim M. (2015). Validation of the hospital anxiety and depression scale in patients with acute low back pain. The Journal of Pain.

[B63-behavsci-15-00040] Walker J. M., Farney R. J., Rhondeau S. M., Boyle K. M., Valentine K., Cloward T. V., Shilling K. C. (2007). Chronic opioid use is a risk factor for the development of central sleep apnea and ataxic breathing. Journal of Clinical Sleep Medicine.

[B64-behavsci-15-00040] Walters A. S., Aldrich M. S., Allen R., Ancoli-Israel S., Buchholz D., Chokroverty S., Coccagna G., Earley C., Ehrenberg B., Feest T. G., Hening W., Kavey N., Lavigne G., Lipinski J., Lugaresi E., Montagna P., Montplaisir J., Mosko S. S., Oertel W., Zucconi M. (1995). Toward a better definition of the restless legs syndrome. Movement Disorders.

[B65-behavsci-15-00040] Weaver T. E., Laizner A. M., Evans L. K., Maislin G., Chugh D. K., Lyon K., Smith P. L., Schwartz A. R., Redline S., Pack A. I., Dinges D. F. (1997). An instrument to measure functional status outcomes for disorders of excessive sleepiness. Sleep.

